# Reduced Surgical Complications After Deceased‐Donor Kidney Transplantation Using Inverted Allograft and Ultrashort Ureteroureterostomy: A Single‐Center Experience

**DOI:** 10.1111/ctr.70235

**Published:** 2025-07-28

**Authors:** Rafael Azevedo Foinquinos, Ana Luiza Souza‐Leão, Ilan Cubits Kyrillos Oliveira Capela, Thales Paulo Batista, Maria Julia Gonçalves Mello, Cristiano Souza Leão

**Affiliations:** ^1^ Department of Urology Instituto de Medicina Integral Professor Fernando Figueira (IMIP) Recife Brazil; ^2^ Medical School Faculdade Pernambucana de Saúde (FPS) Recife Brazil; ^3^ Department of Surgery Universidade Federal de Pernambuco (UFPE) Recife Brazil; ^4^ Department of Clinical Research Instituto de Medicina Integral Professor Fernando Figueira (IMIP) Recife Brazil; ^5^ Department of Surgery Instituto de Medicina Integral Professor Fernando Figueira (IMIP) Recife Brazil

**Keywords:** morbidity, renal transplantation, surgical injuries, urethral stricture, urinary fistula

## Abstract

**Objective:**

To describe the outcomes of combined inverted renal grafts and ureteroureterostomy as the primary operative approach for kidney transplantation (KTx).

**Patients and Methods:**

This case series included adult patients who consecutively underwent deceased‐donor KTx for end‐stage kidney disease at our center between January 2019 and June 2022. All patients received inverted KTx combined with ultrashort anisoperistaltic end‐to‐side ureteroureterostomy, without ureteral stenting. Descriptive analysis focused on the major perioperative outcomes within 90 days post‐transplantation.

**Results:**

Cohort analysis of 211 patients revealed major postoperative complications requiring reoperation in 15 patients (7.11%). Reoperations for urological reasons included four patients (1.90%) with ureteral stricture and ureteral fistula in two patients each (0.95%). Ureteral strictures were managed with pyeloureterostomy, and ureteral fistulas were treated with end‐to‐end ureteral re‐anastomosis, double‐j stenting, JP drain placement, or pyeloureterostomy. Non‐urological complications leading to reoperation included peri‐graft collections and infections such as surgical hematoma (1.42%), deep surgical infection (1.90%), and wound dehiscence (1.90%). Three patients (1.42%) ultimately underwent transplantectomy because of graft loss after severe infection in two patients (0.95%) and acute rejection plus infection in one patient (0.47%). All the remaining patients were treated with surgical exploration, evacuation/irrigation, and wound closure. Vascular complications occurred in one patient with an arterial stricture (0.47%). Delayed graft function was found in 82.9% of the patients, with 95.26% achieving resolution within 4 weeks post‐transplantation.

**Conclusions:**

Inverted KTx combined with ultrashort end‐to‐side ureteroureterostomy is a feasible and safe technique with low rates of urological and vascular complications in our experience. To our knowledge, this is the largest case series using this combined surgical approach as the primary technique for deceased‐donor KTxs.

## 1 Introduction

Kidney transplantation (KTx) is the therapy of choice for improving clinical outcomes in patients with end‐stage kidney disease. Despite the shortage of available organs and risks associated with immunosuppression, evidence supports reduced mortality and a lower risk of cardiovascular events, with a significantly superior quality of life associated with KTx [1, 2]. Furthermore, the relative benefits of transplantation appear to increase over time, even with the observed increase in the age and comorbidities of contemporary transplant recipients [1, 3], which has contributed to transplantation becoming a standard of care and a common surgical procedure worldwide.

From a technical perspective, most KTx procedures are performed with the kidney graft in a craniocaudal position in the iliac fossa, followed by an extravesical ureteroneocystostomy, in which the ureter is anastomosed to the bladder dome and a new anti‐reflux valve is created. However, this approach does not provide a suitable window for venous anastomosis and sometimes encounters challenges owing to the short length of the donor vessels. In these scenarios, the inverted (i.e., upside‐down) graft technique has been proposed to facilitate vessel mobilization because the arterial anastomosis lies distal to the venous anastomosis, which permits venous anastomosis to be performed first, making the procedure easier [4, 5, 6, 7, 8]. Similarly, a few centers have also preferred to perform ureteroureterostomy as an alternative to ureteroneocystostomy in select difficult pediatric KTx cases [9], as a salvage procedure after ureteroneocystostomy [9, 10], or as an alternative to pyeloureterostomy in patients with a completely intra‐hilar renal pelvis during adult KTx [11].

We have previously described our primary option for KTx, combining the inverted technique with ultrashort anisoperistaltic end‐to‐side ureteroureterostomy in adult KTx [12]. This modified approach aims to improve vascular management during anastomosis and enhance blood supply quality using a short ureteral graft while preserving the patient's anti‐reflux system by maintaining the natural orthotopic orifice, potentially reducing the incidence of vesicoureteral reflux and its associated complications. Herein, we present our clinical experience with this combined approach, describing relevant short‐term (i.e., 90‐days) outcomes, such as urological complications and reoperation rates, in a large single‐center cohort of patients who underwent deceased‐donor KTx.

## 2 Materials and Methods

A single‐center case series was conducted on adult patients who underwent deceased‐donor KTx for end‐stage kidney disease at the Instituto de Medicina Integral Professor Fernando Figueira (IMIP). Using a retrospective database populated by one of our postgraduates (i.e., Foinquinos R. A.), patients who received a deceased‐donor KTx combining the inverted technique with ultrashort anisoperistaltic end‐to‐side ureteroureterostomy for treatment of end‐stage kidney disease were selected. The study was limited to adult patients aged between 18 and 70 years who were consecutively operated on between January 2019 and June 2022, and excluded those for whom the main medical records were unavailable. The study protocol was reviewed by the Ethics Research Committee of our institution (CAAE: 58128222.6.0000.5201, acceptance protocol number 5.402.0600; May 11, 2022) and complied with the standards of the current Brazilian ethical guidelines. The requirement for informed consent was waived by the ethics committee because this was an observational retrospective study based on medical chart reviews.

Following the STROBE (Strengthening the Reporting of Observational Studies in Epidemiology) statement (https://www.equator‐network.org/reporting‐guidelines/strobe/), relevant clinical factors such as age, sex, body mass index, comorbidities, type and duration of renal replacement therapy, previous history of KTx, cold ischemia time, duration of postoperative hospitalization, time to dialysis independence (i.e., days from KTx to the last postoperative dialysis), reoperation rates owing to urological and non‐urological reasons, and the presence and severity of delayed graft function were explored. In the current study, prolonged delayed graft function was defined as the requirement for dialysis treatment beyond the first week post‐KTx surgery and severely delayed graft function persisting for >14 days [13, 14, 15]. A detailed description of our institutional approach combining the inverted technique with ultrashort (i.e., 2 cm) anisoperistaltic end‐to‐side ureteroureterostomy has been previously reported [12]. As a routine practice, ureteral stenting was not primarily used during KTx, and abdominal drains were applied selectively at the surgeon's discretion.

During the study period, patients received the same institutional standards of immunosuppressive therapy, including induction with an anti‐interleukin‐2 receptor antibody, calcineurin inhibitor, mycophenolate, and steroids. In patients with no rejection episodes in the first year after transplantation, a progressive dose reduction of steroids was administered to achieve a steroid‐free regimen. The short‐term postoperative follow‐up schedule included daily visits by the surgical team during hospitalization, followed by outpatient clinic visits every 2–4 weeks for at least 90 days post‐surgery. Continuous variables were summarized as medians (interquartile range [IQR]) and categorical variables as frequencies (percentages). Descriptive statistics were calculated using Jamovi v.2.3 (2023), a free open statistical platform available at https://www.jamovi.org.

## 3 Results

**FIGURE 1:**
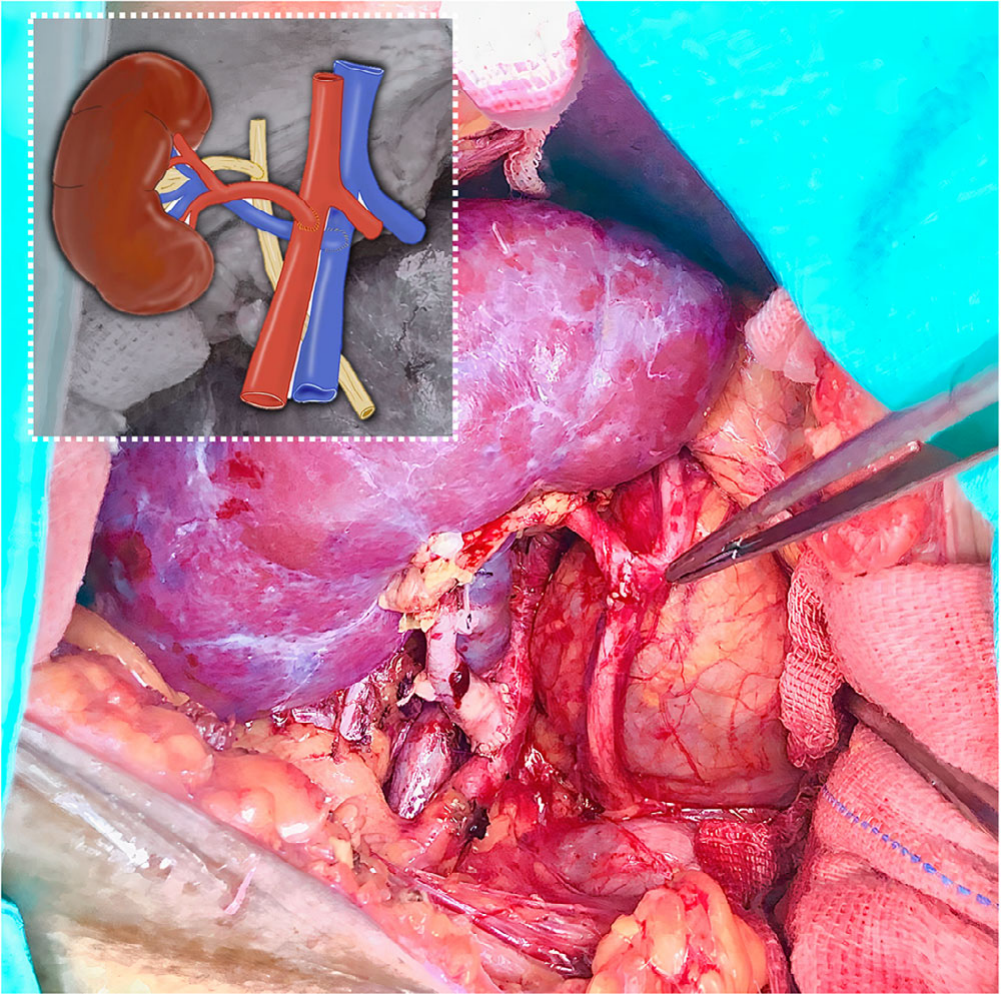
Overview of the procedures combining the inverted approach with an ultrashort end‐to‐side ureteroureterostomy. The ureteral anastomosis is indicated by the surgeon using tissue forceps.

**FIGURE 2:**
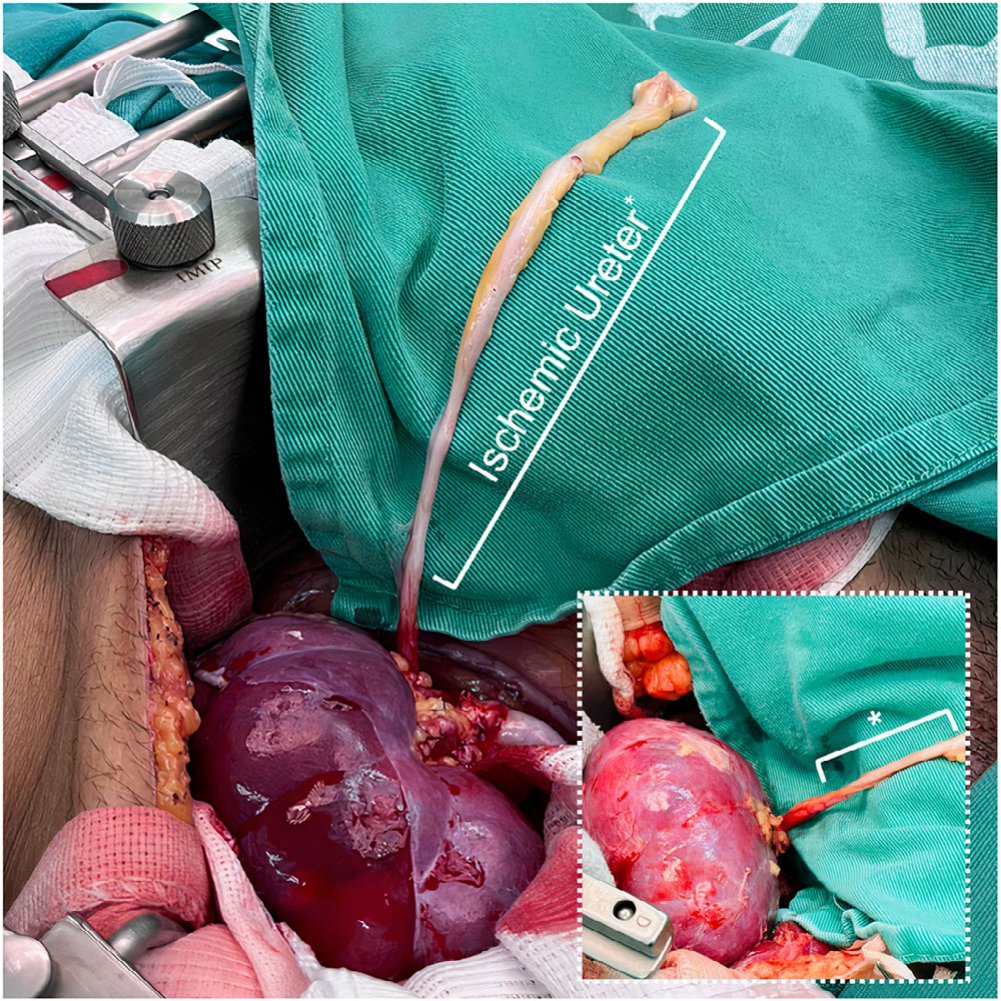
The perfusion advantages of using the short ureteral graft segment to achieve a well‐vascularized uretero‐ureteral anastomosis. This image highlights that most of the ureter is ischemic and only a short proximal segment remains well vascularized after completing the vascular anastomoses.

In this 3‐year case series study, 246 patients who underwent KTx at our institution were screened, 211 of whom were diagnosed with end‐stage kidney disease and met the inclusion criteria. All patients in this cohort received a deceased‐donor kidney graft under general anesthesia with a transversus abdominis plane (TAP) block using an inverted renal graft combined with an ultrashort ureteroureterostomy without ureteral stenting. The median waiting list time under replacement therapy was 3.7 years (IQR, 2.2–6.5 years), and the median cold ischemia time was 25 h (IQR, 17–31 h). Twenty patients (9.47%) had medical records of previous urological diseases, including nephrolithiasis, vesicoureteral reflux, neurogenic bladder, previous unilateral nephrectomy, and ureteral strictures. The baseline demographic characteristics of the patients are summarized in Table 1. Figures 1 and 2 present an overview of the procedures and highlight the perfusion advantages of using a short ureteral graft segment to achieve a well‐vascularized uretero‐ureteral anastomosis, respectively.

**TABLE 1:** Baseline demographics of patients.

Variables	*n* (%) or median (*IQR*)
Age (years)	42.5 (33.4–52.8)
Body mass index (kg/m^2^)	23.5 (20.6–26.2)
Sex	
Male	147 (69.70)
Female	64 (30.30)
Cold ischemia time	
<12 h	19 (9)
12–24 h	72 (34.12)
≥24 h	116 (54.97)
Missed	4 (1.89)
Comorbidities	
Hypertension	110 (52.1)
Diabetes	31 (14.7)
Systemic lupus erythematous	1 (0.50)
Replacement therapy	
Peritoneal dialysis	1 (0.48)
Hemodialysis	210 (99.52)
Previous kidney transplantation	
Yes	9 (4.26)
No	202 (95.74)

Fifteen patients (7.11%) had postoperative complications that required reoperation for urological and non‐urological reasons. Four patients (1.90%) underwent reoperations for urological reasons, including ureteral stricture managed with pyeloureterostomy in two patients (0.95%) and ureteral fistula in two patients (0.95%), treated by end‐to‐end ureteral re‐anastomosis and double‐J stenting plus peri‐anastomotic drainage (JP drain), or pyeloureterostomy. Peri‐graft collections and infections, including surgical hematoma in three patients (1.42%), deep surgical infection in four patients (1.90%), and wound dehiscence in four patients (1.90%), were the most common non‐urological perioperative complications leading to reoperation. Three patients (1.42%) ultimately underwent transplantectomy because of graft loss after severe infection in two patients (0.95%) and acute rejection plus infection in one patient (0.47%). All the remaining patients underwent surgical exploration for evacuation/irrigation and wound closure.

The median hospitalization duration was 11 days (IQR, 8–17 days), and the 90‐day mortality rate in this cohort of deceased‐donor KTx patients was 0.47% (*n* = 1). One patient died from an arterial stricture, which was scheduled for endovascular management, that led to a hemorrhagic complication from central line placement for hemodialysis. In summary, the median time to dialysis independence was 4 days (IQR, 1–10 days), with delayed graft function in 82.9% of the patients (*n* = 175). Delayed recovery requiring dialysis beyond the first week after KTx was recorded in 32.2% of patients (*n* = 68), and severely delayed graft function persisting beyond 14 days was observed in 12.8% of patients (*n* = 27). Most patients (95.26%, *n* = 201) achieved resolution of delayed graft function within 28 days of deceased‐donor KTx.

## 4 Discussion

This study presents a large case series of deceased‐donor KTx combining the inverted allograft approach with an ultrashort anisoperistaltic end‐to‐side ureteroureterostomy. To our knowledge, this is the largest published study to use this approach as the primary technique for deceased‐donor KTxs. Based on our consecutive patient experience, this technique was safe and was associated with low rates of urological and vascular complications, with a correspondingly low rate of reoperation for urological reasons. Furthermore, despite the prolonged cold ischemia time and consequent high incidence of delayed graft function observed in this cohort, almost all patients achieved full renal function recovery within 4 weeks post‐transplantation.

KTx is the cornerstone of the management of patients with end‐stage renal disease. As a major surgical procedure, surgical morbidity may reach approximately 15.8% [16] depending on patient‐ and transplant‐related factors, with 7.4% of patients having an unplanned return to the operating room within 90 days post‐transplantation [17]. These reoperations often result from vascular complications, such as thrombosis and arterial strictures; urological issues, such as urinary leakage and obstruction of the collecting system; or peri‐graft collections and infections [16, 17, 18]. Of note, vascular complications are found in 2.6% of patients [19, 20], with urological complications ranging from 3.7% to 6.0% in the most recent reports [18], but reaching as high as 16.26% in KTx developed with expanded criteria donors [21]. In our experience, despite a similar rate of postoperative complications requiring reoperation, only 1.90% and 0.47% of the patients had major urological or vascular complications, respectively.

Longer cold ischemia time is a determinant of delayed graft function after KTx [22], which significantly affects long‐term patient outcomes and the occurrence of surgical complications [23]. The incidence of allograft malfunction ranges from 20% to 30% in the United States [24] and nearly 50% in Europe [25], with wide variations among Brazilian centers, ranging from 29.9% to 87.7% [13, 26, 27]. In the current study, despite the high rate of delayed graft function, the median time to dialysis independence was only 4 days, in contrast to a median of twice that reported in previous reports on deceased‐donor KTx [14]. Accordingly, the requirement for dialysis beyond 1 week post‐transplantation was 32.2%, and severely delayed graft function persisting for >14 days occurred in 12.8% of our patients, which contrasts with the rates of 22.5% [15] and 26% [14], respectively, in previous large cohorts. Finally, our patients achieved similarly high rates (95%) of the full resolution of graft function within 28 days post‐transplantation [14], demonstrating that the effect of a long cold ischemia time on graft function was mitigated over time.

In 1994, Ballesteros et al. [28] reported a case series of 174 patients and found no evidence of increased vascular complications associated with inverted grafts. This is consistent with more recent studies describing a vascular thrombosis rate as low as 0.57% [29] with no graft loss owing to vascular or urological reasons [30] associated with this approach. Moreover, inversion of the kidney allograft is a safe and effective technique, with no significant differences in the risk of postoperative adverse events or overall outcomes compared with standard techniques [8, 31, 32]. Similarly, end‐to‐side uretero‐ureteral anastomosis without native ureteral ligation has been successfully used in pre‐emptive kidney transplants and in patients with disused atrophic bladders to reduce urological complications and prevent symptoms or obstructions from native kidneys [33, 34]. However, most of these studies involved small cohorts, making our series the largest reported to date on inverted allografts and end‐to‐side ureteral anastomosis, as well as the first to combine these approaches in consecutive cases.

We chose to apply the same surgical approach uniformly across all procedures to ensure technical consistency and facilitate the standardization of both operative and postoperative management. Even in cases with adequate vascular and ureteral lengths, we favor this technique because of its several intrinsic advantages. First, inverted positioning facilitates vascular anastomosis by allowing a sequential, tension‐free connection, particularly to improve access to the venous structures. Second, preserving the recipient's native anti‐reflux mechanism reduces the risk of vesicoureteral reflux and enables future ureteroscopic access via the preserved native ureteral orifice, offering advantages over ureteroneocystostomy. Third, the use of a shorter ureteral graft decreases the risk of ischemia and promotes better anastomotic healing. Notably, without kidney inversion, the ureter follows a longer, more angulated, and tortuous course, which may increase the likelihood of complications and hinder ureteral anastomosis. These advantages justify the adoption of this method as standard practice, even when anatomical conditions might otherwise favor a conventional approach.

This was a descriptive, non‐comparative study with limited external validity owing to its single‐center design and the inherent biases of retrospective analyses. In this setting, we focused on major surgical outcomes that are usually well‐documented in medical charts and are of great interest in clinical practice. Another limitation of our study was the prolonged cold ischemia time and the unavailability of perfusion machines in the context of the public health system in our developing country. On the other hand, this is the largest series to date of KTx using the inverted approach, and the pioneering study involving its combination with an ultrashort end‐to‐side ureteral anastomosis as the primary method for KTxs.

Inverted renal allografts are currently used to address specific anatomical and surgical challenges, particularly when faced with a short right renal vein following a right living‐donor nephrectomy [5, 6, 8]. Similarly, most transplantation centers only use ureteroureterostomy to overcome technical issues and treat post‐transplant complications [9, 10, 11, 33]. Here, we have outlined a surgical technique that combines both approaches, providing some basis for planning future prospective studies to directly compare this approach with other standard surgical techniques in KTx. This technique is a reproducible method for achieving secure and comfortable vascular anastomosis without requiring complex surgical maneuvers. It also facilitates ureteroscopic management of native or implanted kidneys via the preserved natural orifice of the recipient's antireflux system, with the only formal contraindication being a history of grade III‐V vesicoureteral reflux with recurrent infections.

## 5 Conclusions

Inverting renal allografts and performing ultrashort ureteroureterostomy appear to be feasible primary approaches for deceased‐donor KTx. In our experience, this combined technique was associated with low rates of urological and vascular complications. Further randomized controlled studies are necessary to validate whether this technique offers advantages over standard approaches.

## Author Contributions

**Rafael Azevedo Foinquinos**: formal analysis, investigation, and data curation. **Ana Luiza Souza‐Leão**: investigation, data collection, and writing—original draft. **Ilan Cubits Kyrillos Oliveira Capela**: investigation, data collection, and writing—original draft. **Thales Paulo Batista**: conceptualization, methodology, formal analysis, investigation, writing—original draft and review and editing, and visualization. **Maria Julia Gonçalves Mello**: formal analysis, investigation, writing—original draft, and review and editing. **Cristiano Souza Leão**: formal analysis, investigation, writing—original draft and review and editing, visualization, and project administration and supervision.

## Conflicts of Interest

The authors declare no conflicts of interest.

## Statement on the Use of Generative Artificial Intelligence

The authors declare the use of artificial intelligence, specifically the free service LetsEnhance (https://letsenhance.io/pt/), to enhance the quality of images captured during surgical procedures using their own smartphones.

## Data Availability

We have no plan to make individual participant data available to other researchers since data sharing was not required in the study protocol initially reviewed and approved by our Ethics Research Committee (Institutional Review Boards).
